# Expression of circadian clock genes and proteins in urothelial cancer is related to cancer-associated genes

**DOI:** 10.1186/s12885-016-2580-y

**Published:** 2016-07-27

**Authors:** Jorunn Litlekalsoy, Kari Rostad, Karl-Henning Kalland, Jens G. Hostmark, Ole Didrik Laerum

**Affiliations:** 1 Department of Clinical Science, The Gade Laboratory of Pathology, University of Bergen, Bergen, Norway; 2 Department of Clinical Medicine, Section of Surgery, University of Bergen, Bergen, Norway; 3 Institute of Biomedical Laboratory Sciences and Chemical Engineering, Bergen University College, Bergen, Norway; 4 Department of Microbiology, Haukeland University Hospital, Bergen, Norway; 5 Department of Urology, Surgical Clinic, Haukeland University Hospital, Bergen, Norway; 6 Department of Pathology, Haukeland University Hospital, Bergen, Norway; 7 Department of Clinical Science, Haukeland University Hospital, N-5021 Bergen, Norway

**Keywords:** Circadian clock genes, Casein kinases, Oncogenes, Tumour suppressor genes and cytokeratins

## Abstract

**Background:**

The purpose of this study was to evaluate invasive and metastatic potential of urothelial cancer by investigating differential expression of various clock genes/proteins participating in the 24 h circadian rhythms and to compare these gene expressions with transcription of other cancer-associated genes.

**Methods:**

Twenty seven paired samples of tumour and benign tissue collected from patients who underwent cystectomy were analysed and compared to 15 samples of normal bladder tissue taken from patients who underwent cystoscopy for benign prostate hyperplasia (unrelated donors). Immunohistochemical analyses were made for clock and clock-related proteins. In addition, the gene-expression levels of 22 genes (clock genes, casein kinases, oncogenes, tumour suppressor genes and cytokeratins) were analysed by real-time quantitative PCR (qPCR).

**Results:**

Considerable up- or down-regulation and altered cellular distribution of different clock proteins, a reduction of casein kinase1A1 (*CSNK1A1*) and increase of casein kinase alpha 1 E (*CSNK1E*) were found. The pattern was significantly correlated with simultaneous up-regulation of stimulatory tumour markers, and a down-regulation of several suppressor genes. The pattern was mainly seen in aneuploid high-grade cancers. Considerable alterations were also found in the neighbouring bladder mucosa.

**Conclusions:**

The close correlation between altered expression of various clock genes and common tumour markers in urothelial cancer indicates that disturbed function in the cellular clock work may be an important additional mechanism contributing to cancer progression and malignant behaviour.

**Electronic supplementary material:**

The online version of this article (doi:10.1186/s12885-016-2580-y) contains supplementary material, which is available to authorized users.

## Background

Time is a fundamental part of all biological processes in tissues and cells. Both in rodents and humans, the circadian timing system affects many cellular and physiological functions, including cell proliferation, metabolic pathways, protein synthesis and energy metabolism [1]. Severe and prolonged disturbances of the circadian timing system are believed to predispose to cancer development in different organs, not only in the mammary and prostate glands, but also in several other types of cancer, including ovarian, kidney, brain, colorectal, lung, head/neck, pancreatic cancer and hematological malignancies [2–14].

The mammalian circadian clock system consists of positive and negative regulators, with a complex auto-regulatory transcriptional and translational feedback program. By accumulating and binding to the promoter region of the two transcriptions factors, BMAL1 and CLOCK, PER and CRY proteins reduce the transcription of many genes, including their own. This occurs during ambient light exposure via the master clock in the brain, the suprachiasmaticus nucleus (SCN). The corresponding proteins oscillate with a delayed phasing and with maximum levels at dusk [15].

The transcription factors CLOCK and BMAL1 form a heterodimer which in humans is acting stimulatory on gene transcription during night time. CLOCK also contributes to chromatin-remodelling and mediates acetylation of BMAL1. The type of phasing can vary from organ to organ. For instance, BMAL1 undergoes rhythmic acetylation in the liver where the timing parallels the down-regulation of circadian transcription in clock-controlled genes.

The 24 h clock generation is modified by post-translational events such as phosphorylation and ubiquitination which contribute to precision, stability and nuclear translocation of the core clock proteins. PER and BMAL1 have also been identified as tumour suppressors [15–20]. Casein kinase 1 epsilon and delta (CSNK1E and CSNK1D) are critical in regulating the core circadian protein turnover in mammals. Mutations in either of these kinases may thus have dramatic effects on the circadian period [21].

Urothelial carcinoma of the bladder is a very complex malignancy with multiple alterations in complementary pathways. The advent of high-throughput methods of molecular analysis, as microarray-based approaches, has been used extensively to look for expression profiles in effort to sub-classify bladder cancer (stage and pathways) and to predict outcomes and response to systemic treatments. Several tissue and blood-based biomarkers have been identified, but status as of today is that no biomarker panel is yet validated for individual prognostic and daily clinical practice. A problem is that most researchers combine biomarkers from a single pathway (cell-cycle, apoptosis or angiogenesis) while the focus rather should be in investigating biomarker combinations that encompass a variety of different pathways to increase the predictive value and opportunity for targeted treatment. Standard pathological features and imaging are insufficient to allow accurate staging, prognostication and prediction of the patient’s outcome [22, 23]. This reveals an urgent need for identifying novel biomarkers that can define the invasive urothelial carcinomas with intrinsic property for recurrence and metastases.

The urinary system undergoes significant circadian rhythms in humans. During day and night both urinary excretion and extrusion of urine are actively regulated by several internal factors, such as antidiuretic hormone [24]. Such circadian variations led us to postulate that similar to other organs, perturbation of the clockwork may be a contributory mechanism of dysregulation during the development of urothelial cancer. Since clock genes have a modifying role in the gene regulation, they may interact with the transcription of oncogenes and/or tumour suppressor-genes. If so, they might be used as independent or additional markers of malignant behaviour. Therefore, ten key proteins of the clockwork were selected for a combined analysis of transcriptional activity and presence of their proteins in the malignant cells. For comparison, simultaneous analyses of gene-expression patterns were performed for oncogenes and suppressor-genes that are commonly altered in urothelial cancer.

## Methods

### Patient material and tissue

Twenty-seven patients with invasive urothelial cancer undergoing cystectomy from 2006 to 2009 were included. General procedures for the cystectomy patients are that the patients enter the operating room around 07:45 in the morning. The anesthesia is completed around 08:20 and within the next 5–10 min open surgery is performed. The bladder is removed from the body around 10:00 whereupon the surgeon immediately collects tissue samples from tumour and adjacent normal appearing mucosa into separate tubes. Within twenty minutes, the harvested bladder biopsies are cut into small pieces and snap frozen at −80 °C. Patient details are given in Table 1. Normal bladder biopsies were taken from 15 male patients who had TUR-P (transurethral resection of the prostate) for benign prostatic hyperplasia (BPH). The mucosal biopsies consisted of the whole urothelial layer and some underlying connective tissue. A major part of the cell nuclei were from urothelium as compared to sub-mucosal fibroblasts. Both the cystectomies and the unrelated normal mucosa were harvested in the time period 9 to 12 AM. Paraffin-embedded tissue slides were made for histological diagnostics, and classified by the WHO and NM-system. The study was approved by the Regional Ethical Committee (REK No. 12226/REK No. 2009/1527).

**Table 1:** Tumour grade, invasiveness, T-stage, ploidy and survival in the individual patients

No	G	V.I.	pTa	pT1	pT2A	pT2B	pT3B	D	A	D-S	A-S	Survival A/D
1	Low				x			x		4,22		A
2	High	x				x			x	7,25	1,9	D/1m
3	High			x					x	-	17	D/10m
4	Low				x			x		7,7		A
5	High	x				x		x		8		A
6	Low		x					x		3,5		A
7	High				x				x	12,89	23,04	D/6m
8	Low		x					x		-	-	A
9	High	x			x				x	72	10,06	A
10	High		x						x	7,91	21	D/10m
11	Low			x				x		7,2		A
12	High				x				x	8,13	26,2	D/14m
13	Low		x						x	6,86	-	A
14	High		x						x	8,2	32	A
15	High	x			x				x	6,2	28	A
16	High	x			x				x	12	21	A
17	High	x	x						x	14	20	A
18	Low	x			x				x	4,4	-	D/11m
19	High	x			x				x	64	15	A
20	High	x			x				x	24	18	D/17m
21	High	x					x		x	18	18	A
22	High				x				x	84	20	D/3m
23	Low	x		x				x		18	17	A
24	High						x		x	41	38	D/10m
25	Low			x					x	41	15	A
26	Low	x			x			x		3,3		D/24m
27	High				x				x	13	20	D/10m

*No* case number, *G* grade, *V.I*. vascular invasion, *pTa-pT1-pT2A-pT2B-pT3B* tumour stage, *D* Diploid, *A* Aneuploid, *D-S* Diploid S-phase, *A-S* Aneuploid S-phase, *Survival A/D* survival after surgery (in months, m), *A* alive, *D* dead

### Immunohistochemistry

The paraffin blocks were cut in 5 μm sections and stained with antibodies listed in Table 2. The sections were de-paraffinised and pre-treated as listed in Table 2, and stained as described earlier [25]. Sections of tissue microarrays made of twelve different tissues, reported to express one or more of our chosen proteins, served as control.

**Table 2:** Specifications of antigens and corresponding antibodies

Antigen	Specificities	Purchaser	Dilution	Pre-treatment
PER1 (Per12-A)	Polyclonal	AH Diagnostics AS Fjellgata 1, Oslo	1:50, overnight at 4 °C	Microwave treatment for 10 min at 750 W and 20 min at 500 W in 10 mmol/L citrate buffer pH6
PER2 (N-19, sc-7728)	Polyclonal	Santa Kruz Biotecnology Inc. Europe	1:200, overnight at 4 °C	Microwave treatment for 10 min at 750 W and 20 min at 500 W in 10 mmol/L citrate buffer pH6
PER3 (Per32-A)	Polyclonal	AH Diagnostics AS Fjellgata 1, Oslo	1:50, overnight at 4 °C	Microwave treatment for 10 min at 750 W and 20 min at 500 W in 10 mmol/L citrate buffer pH6
CRY1 (W-L5, sc-101006)	Monoclonal	Santa Kruz Biotecnology Inc. Europe	1:200, overnight at 4 °C	Microwave treatment for 10 min at 750 W and 20 min at 500 W in 10 mmol/L citrate buffer pH6
CRY2 (P-21, sc-130731)	Polyclonal	Santa Kruz Biotecnology Inc. Europe	1:200, overnight at 4 °C	Microwave treatment for 10 min at 750 W and 20 min at 500 W in 10 mmol/L citrate buffer pH6
BMAL 1 (LS-B660/12275)	Polyclonal	Lifespan Biosciences (Nordic biosite)	1:100, overnight at 4 °C	Microwave treatment for 10 min at 750 W and 20 min at 500 W in 10 mmol/L citrate buffer pH6
CLOCK (LS-B278/18928	Polyclonal	Lifespan Biosciences (Nordic biosite)	1:500, overnight at 4 °C	Microwave treatment for 10 min at 750 W and 20 min at 500 W in 10 mmol/L citrate buffer pH6
Anti-CSNK1α1L	Polyclonal	Abcam.com England	1:150, overnight at 4 °C	Microwave treatment for 10 min at 750 W and 20 min at 500 W in 10 mmol/L citrate buffer pH6
Casein kinase 1Ɛ (Sc-25423)	Polyclonal	Santa Kruz Biotecnology Inc. Europe	1:100, overnight at 4 °C	Microwave treatment for 10 min at 750 W and 20 min at 500 W in 10 mmol/L citrate buffer pH6
Casein kinase 1α (Sc-28886)	Polyclonal	Santa Kruz Biotecnology Inc. Europe	1:100, overnight at 4 °C	Microwave treatment for 10 min at 750 W and 20 min at 500 W in 10 mmol/L citrate buffer pH6

#### Evaluation of staining results

The analyses were made separately for the tumour and neighbouring benign tissue from cystectomies, and unrelated normal mucosa. Positive staining of epithelial cells was estimated as weakly, moderately and strong, (separately for the nucleus (N) and the cytoplasm (C)). Counting was performed on cells from tumour, normal appearing mucosa without atypia, and normal mucosa from the 15 individuals (Table 3). For control, the same staining procedure was performed on tissue microarrays comprising other human tumours/normal tissues. All cases were scored on coded specimens separately by ODL and JGH.

**Table 3:** Mean scores of positivity in nucleus and cytoplasm for the clock proteins

Protein	Cancer cells		Neighbouring mucosa		Normal mucosa	
	Nucleus	Cytopl.	Nucleus	Cytopl.	Nucleus	Cytopl.
PER 1	2.17	0	1.71	0	2.00	0
+/− SEM	0.15	0	0.10	0	0	0
PER 3	0.22	0.43	0.67	0.73	0	1.15
+/− SEM	0.08	0.11	0.13	0.15	0	0.13
CRY 1	2.08	1.96	1.84	1.27	1.76	0.62
+/− SEM	0.16	0.11	0.14	0.20	0.13	0.15
CRY 2	0	0.83	2.31	2.75	2.00	2.00
+/− SEM	0	0.13	0.23	0.18	0.26	0.28
BMAL1	1.42	2.40	2.33	2.27	1.16	2.08
+/− SEM	0.20	0.12	0.19	0.20	0.21	0.20
CLOCK	2.04	2.23	2.57	2.52	2.75	2.91
+/− SEM	0.16	0.12	0.13	0.13	0.14	0.09
Casein kinase 1 alpha						
	1.93	2.70	2.78	3.00	2.90	3.00
+/− SEM	0.18	0.10	0.13	0.00	0.11	0.00
Casein kinase 1 alpha 1 L						
	1.96	2.59	2.88	2.96	2.54	2.92
+/−SEM	0.24	0.12	0.08	0.04	0.22	0.08
Casein kinase 1 epsilon						
	2.93	2.07	2.75	1.95	3.00	2.00
+/− SEM	0.05	0.05	0.10	0.09	0	0

+/− SEM: +/− standard error of the arithmetic means

### Flow cytometry (FCM)

FCM was performed on single cell suspensions of tumour tissue obtained by cutting the tissue into small pieces which were shaken, filtered, spun down, re-suspended in PBS and fixed by addition of 96 % ethanol, stained with propidium iodide as earlier described [26] and analysed on a FACScan flow cytometer (Becton Dickinson, Palo Alto, CA, USA). Normal human lymphocytes were used as standard, and the ploidy index (PI) was calculated as a ratio between the peak channel for the tumour cells and the peak channel for the lymphocytes.

### RNA extraction and real-time quantitative PCR (qPCR)

#### RNA purification and single-stranded cDNA synthesis

Biopsies were ground to powder under liquid N2. Total RNA was extracted according to standard protocols (Invitrogen Trizol LS protocol and Qiagen miRNeasy protocol; Invitrogen, Carson City, CA). 30 μl of single-stranded cDNA for qPCR analysis was synthesised from 1 μg of total RNA according to Ambion (Ambion, TX, USA) instructions.

#### Endogenous control and endogenous control cards

The different tissue types included in our study were initially studied with respect to gene expression of 16 different housekeeping genes, to assess which one was best suited as endogenous control for our purpose. Two endogenous control cards accommodating 8 samples each, in triplicate, were applied. β-actin (ACTB) proved to be the most suitable endogenous control for our three tissue types and therefore chosen when designing the Taqman low density arrays (TLDA) cards. In addition GAPDH was added in the TLDA cards as standard (from the supplier), but was not used in our further calculations.

#### Real-time quantitative PCR (qPCR) in low-density array format

Taqman low density arrays (TLDA) are customizable, 384-well microfluidic cards for real-time qPCR (Applied Biosystems (ABI)). Each TLDA card was configured for 24 genes in duplicates, including β-actin and GAPDH as endogenous controls, core clock-genes and genes encoding several tumour markers (TaqMan assays are listed in Table 4). Single-stranded cDNA corresponding to 200 ng of total RNA was diluted in Taqman Universal buffer (ABI) and added to each loading well. The samples were distributed to the micro wells by centrifugation for 1 min at 343xg. The cards were placed in an ABI PRISM 7900HT Sequence Detection System thermocycler for 40 cycles: 15 s at 95 °C and 60 s at 60 °C. The SDS2.3 and RQ manager 1.2 software (ABI) were used for analysis and data were exported to Excel for further visualization. Data Assist v.3.01 (ABI) was utilized for hierarchical cluster analysis and generation of correlation plots. The gene expression data were analysed using the comparative Ct-method (∆∆Ct). Gene expression levels were normalized against ß-actin and calibrated against a chosen calibrator to provide fold change relative gene expression levels. Two separate gene expression analysis were performed in order to study the relative differential gene expression (fold change (Relative quantity (RQ)) in the respective tissues: tumour and neighbouring benign tissue relative to unrelated normal mucosa, and relative gene expression levels in tumour *versus* neighbouring mucosa.

**Table 4:** List of TaqMan gene expression assays and their corresponding proteins

Gene assay	Protein	Gene assay	Protein	Gene assay	Protein
Hs00978050_m1	H-RAS	Hs01034249_m1	p53	Hs00242988_m1	PER 1
Hs00364284_m1	K-RAS	Hs00923894_m1	p16	Hs00256143_m1	PER 2
Hs00180035_m1	N-RAS	Hs02621230_m1	pTEN	Hs00213466_m1	PER 3
Hs01076078_m1	EGFR	Hs00559840_m1	Cytokeratin 7	Hs01565974_m1	CRY 1
Hs00182181_m1	uPAR	Hs00196158_m1	Cytokeratin 1	Hs00323654_m1	CRY 2
Hs01126606_m1	PAI 1	Hs00361185_m1	Cytokeratin 5	Hs00154147_m1	BMAL 1
		Hs00166289_m1	Cytokeratin10	Hs00231857_m1	CLOCK
Hs99999905_m1	GAPDH	Hs00265033_m1	Cytokeratin14	Hs01887794_m1	CK1A1L
				Hs00793391_m1	CK1A1
				Hs00266431_m1	CK1ε

### Statistics

Statistical Package for the Social Sciences (SPSS v.12) (SPSS Inc. Chicago, Illinois) was utilized for statistical analysis. The Spearman’s rank correlation (correlations coefficient, c) was used to determine significant correlation between the various gene expressions. The Mann-Whitney non-parametric rank test was used to identify correlation between the gene expressions in the tumours compared to neighbouring mucosa. Data Assist v.3.01 (ABI) was applied on the gene expression data to calculate Pearson’s product monument correlation coefficients (r) for each sample represented in the various tissue types. Pearson’s correlation was used for the hierarchical cluster analysis and generation of heat maps of gene expression. Data Assist v.3.01 (ABI) performed a two-sample, two-tailed Student’s t-test for comparing the fold change values (2^(−deltaCt)^) of the separate biological groups (normal bladder mucosa, neighbouring benign and tumour tissue), and a p-value was calculated. The results were presented in the mRNA fold change gene expression plots (log fold change *versus* sample group).

## Results

### Immunohistochemistry

#### Stimulatory clock proteins/casein kinases

Cytoplasmic BMAL1 staining was slightly stronger in the tumour and the neighbouring mucosal cells than in the normal, unrelated mucosa. In the nuclei, BMAL1 was significantly increased in neighbouring tissue, and also slightly increased in tumour tissue compared to normal mucosal cells (Table 3). Six cases expressed neither BMAL1 nor CRY2 in the nucleus. When this was compensated for, the remaining positive cases for BMAL1 had a mean score in the nucleus of 1.84 +/− SEM 0.15, which is significantly higher than in the normal mucosa. CLOCK was significantly reduced in the tumour cells, but not in the nucleus or cytoplasm in the neighbouring mucosa.

Casein kinase 1A and 1A1Like were both significantly reduced in the tumour nuclei, but not in the cytoplasm. Casein kinase 1E was equally expressed in both nucleus and cytoplasm.

#### Inhibitory clock proteins

PER1 was positive in the nucleus and absent in cytoplasm of neoplastic, neighbouring and normal mucosa (Table 3). PER2 did not give satisfactory staining and was omitted. PER3 was absent in nucleus of normal mucosa, but expressed in cancer cells and their neighbouring mucosa. Opposite, it was lower in the cytoplasm of cancer cells and neighbouring tissue compared to normal mucosa, and there seemed to be a significant shift from cytoplasm to nucleus in malignancy. CRY1 was significantly increased in tumour cytoplasm and neighbouring mucosal cells. The increased expression of CRY1 in the cancer cells was three times higher than in normal mucosa. CRY2 was absent in the nucleus in cancer cells and low in the cytoplasm, while neighbouring and normal mucosal cells showed no major differences.

Altogether, this indicates complex alterations, where the main features were redistribution between nucleus and cytoplasm, and an increase of both stimulatory and inhibitory clock proteins, see in Additional file 1: Figure S1.

### Gene expression analysis

#### Raw data and general pattern

**Fig. 1:**
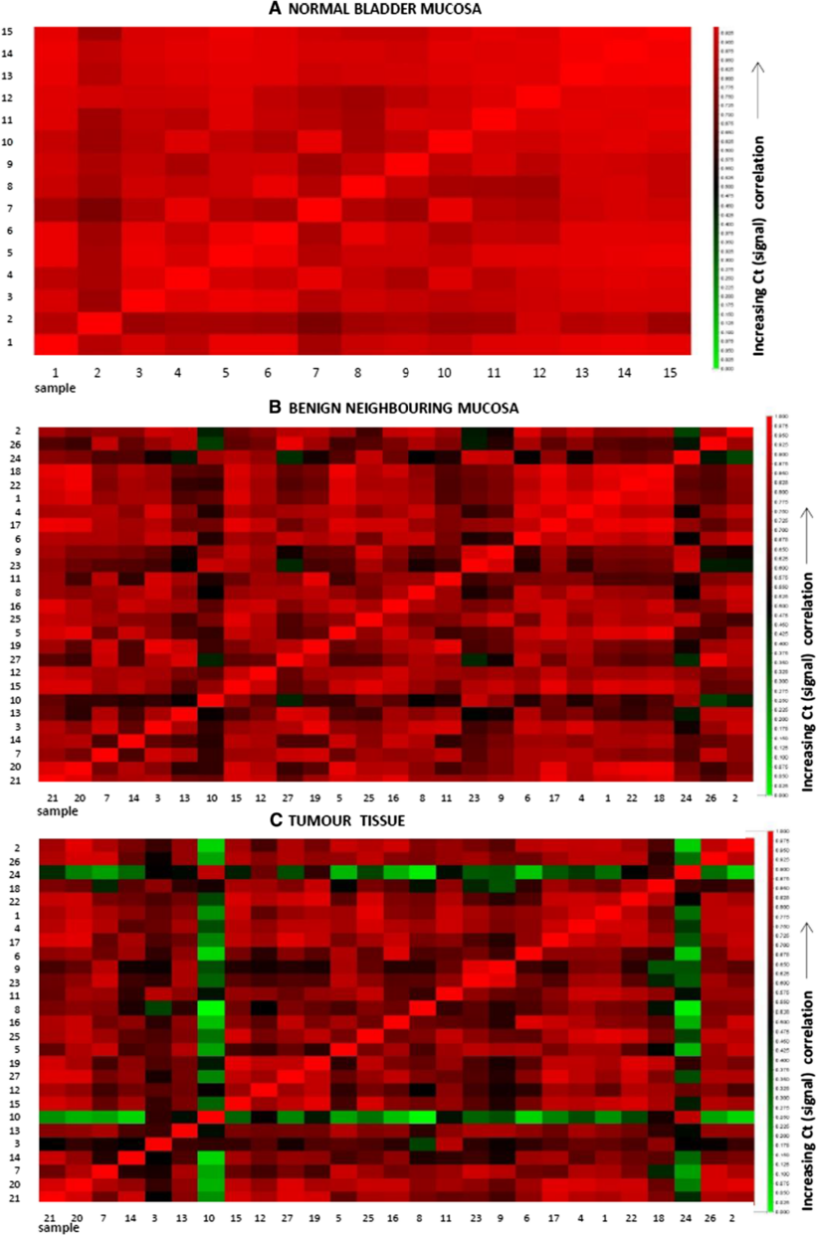
Gene expression signal correlation plots. The plots display the correlations between mRNA normalized gene expression levels in the normal control bladder tissue samples of 15 patients with BPH (**a**), benign tissue peripheral to the tumour (**b**) and tumour tissue (**c**) of 27 cystectomy patients, respectively. Pearson’s product moment correlation coefficients (r) for each pair of samples were calculated using DataAssist from Applied Biosystems. Each cell represents a different scatter plot, coloured to indicate the strength of the correlations between the samples. The higher the correlation between the gene expression levels in the two samples (the closer the correlation coefficient (r), is to 1), the colour moves towards brighter red. The poorer the correlation between the gene expression levels in the two samples (the closer r is to 0), the colour moves towards darker red and then green, indicating no correlation. All samples are correlated with each other for each of the selected genes

**Fig. 2:**
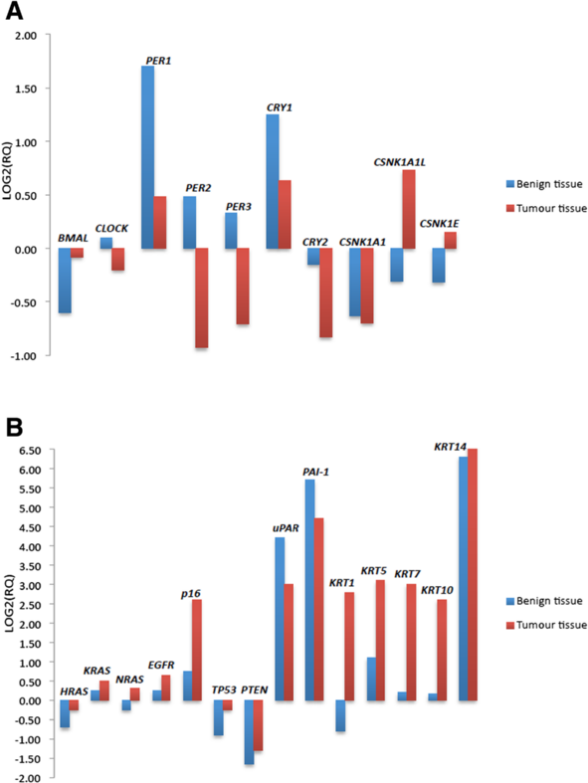
mRNA fold change gene expression plots. Gene expression levels in benign neighbouring mucosa and tumour tissue relative to normal bladder mucosa tissue from BPH patients. The relative quantity plots display the log2 fold change in mRNA levels in the benign (*blue bars*) and tumour (*red bars*) tissue taken from cystectomies (27 patients) versus normal bladder tissue from BPH patients. The *bars* in **a**. display the log2 fold change (log2 RQ) in mRNA levels of the clock genes, while the tumour marker genes are plotted in **b**. Genes with a negative value are down-regulated, while genes with a positive value are up-regulated in the malignant bladder (tumour and benign tissue) versus the normal bladder (whose log2 value is 0 for each gene). Statistical significance with a p-value ≤ 0.05 was found for *KRT7, PER1, PER2, PTEN, uPAR* and *PAI-1* (Two-sample, two-tailed Student’s t-test)

**Fig. 3:**
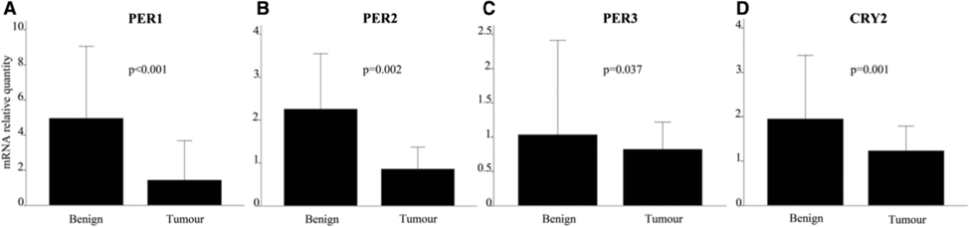
Relative mRNA quantity of *PER1*, *PER2*, *PER3* and *CRY2*. Real-time quantitative PCR expression levels normalized against the endogenous control *β-actin (ACTB).* The figure gives the comparison between 27 tumour and matched benign bladder tissue samples. Columns, median; bars, **a**: *PER1*, **b**: *PER2*, **c**: *PER3* and **d**: *CRY2*. The relative gene expression of all four genes was significantly elevated in the benign versus malignant bladder tissue. The changes were consistent for each pair of tumour - neighbouring mucosa, indicated by the *p*-value of the statistical test (non-parametric paired samples Mann-Whitney test)

**Fig. 4:**
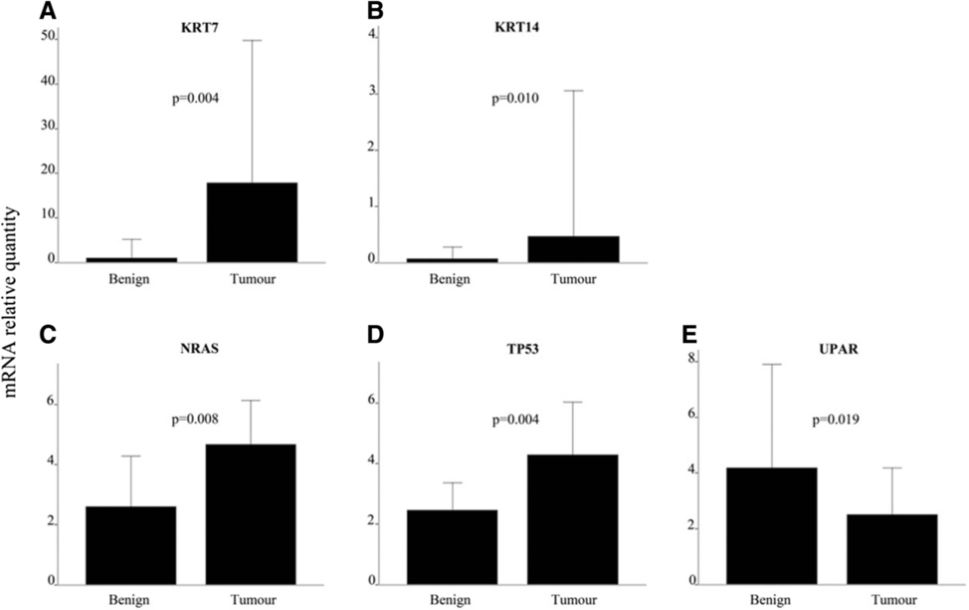
Relative mRNA quantity of *KRT7*, *KRT14*, *NRAS*, *TP53* and *UPAR*. Real-time quantitative PCR expression levels normalized against the endogenous control *β-actin.* The figure gives the comparison between 27 tumour and matched benign bladder tissue samples. Columns, median; bars, **a**: *KRT7*, **b**: *KRT14*, **c**: *NRAS*, **d**: *TP53* and **e**: *UPAR*. The gene expression levels of the cytokeratins, the *NRAS* and *TP53* were significantly elevated in the tumour versus benign bladder tissue, while the expression of *UPAR* was significantly elevated in the benign tissue compared to the tumour. The changes were consistent for each pair of tumour - neighbouring mucosa, indicated by the p-value of the statistical test (non-parametric paired samples Mann-Whitney test)

**Fig. 5:**
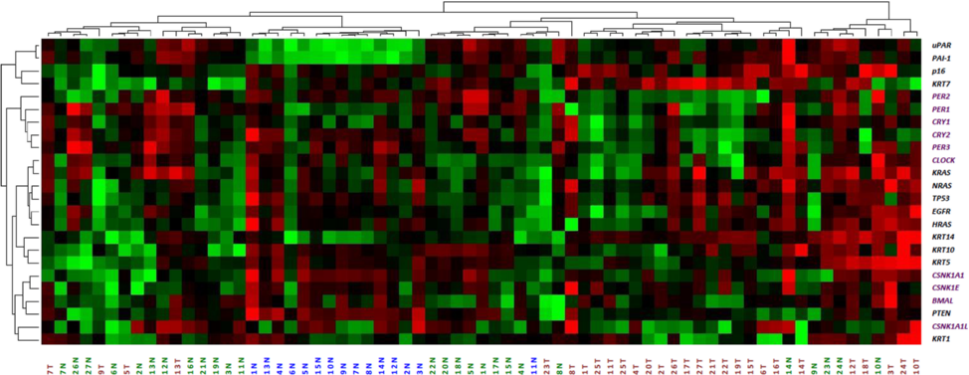
Unsupervised hierarchical cluster analysis of differentially expressed genes. Normal bladder tissue from 15 unrelated donors with BPH together with tumour and matched benign tissue from 27 cystectomy patients were analysed. Real-time qPCR expression data were imported into DataAssist (ABI) for unsupervised hierarchical cluster analysis. Distances between samples and assays were calculated based on the delta-Ct values using Pearson’s correlation. Differentially expressed genes are represented in rows and the different samples are represented in columns. Each cell in the heat map represents one samples relative expression of one gene. For each gene assay, the middle expression level was set as the median of all of the delta-Ct-values of all samples for that gene assay. Gene expression colour codes in the heat map: *Green colour* represents relative levels of mRNA lower than the middle value for that gene expression assay (decreased gene expression); *Red colour* represents levels of mRNA higher than the middle expression level (increased gene expression); *Dark colour* reflects an mRNA expression level closer to the middle expression level (no major increase or decrease in gene expression). Patient samples: *Blue colour*: The normal bladder tissue taken from BHP patients is numbered 1–15N; *Green colour*: Normal benign tissue taken peripherally to the tumour is numbered 1–27N; *Red colour*: tumour tissue is numbered 1–27T. Genes: *Purple colour*: Clock genes; *Black colour*: cancer associated genes. (Clustering method: complete linkage. Map type: assay centric)

The over-all differences in gene expression pattern in tumours compared to matched neighbouring mucosa are shown in Table 5. The gene-expression signal correlation plot is visualized in Fig. 1. The mRNA fold change in tumour and neighbouring mucosa from 27 patients relative to normal mucosa from 15 unrelated donors are visualized in Fig. 2. Figures 3 and 4 display relative quantity of mRNA in tumour compared to neighbouring mucosa of 27 patients for the genes found statistically significant. Figure 5 shows a hierarchical cluster diagram (heat map) of differential expression of 22 genes in normal mucosa from 15 unrelated donors together with tumour/neighbouring mucosa from 27 patients (cystectomies).

**Table 5:** Relative gene expression levels of clock genes and common tumour markers from cystectomies (Tumour/Benign-fold change)

A. Relative mRNA gene expression levels of clock genes and common tumour markers from cystectomies (Tumour/Benign-fold change)																								
	GENES																							
Patient sample	BMAL	CLOCK	PER1	PER2	PER3	CRY1	CRY2	CSNK1A1L	CSNK1A1	CSNK1E	TP53	p16	PTEN	EGFR	HRAS	KRAS	NRAS	Upar	PAI-1	KRT7	KRT1	KRT5	KRT10	KRT14
1	1,3	0,8	0,1	0,1	0,3	0,5	0,3	34,8	0,6	0,5	0,7	8,1	0,5	0,8	0,9	0,6	0,9	0,1	0,1	0,5	1,1	0,0	0,1	9,5
2	2,1	1,0	0,9	1,2	0,8	1,5	1,3	0,0	1,3	3,5	2,0	6,3	2,4	0,8	1,9	1,0	2,3	1,6	0,6	83*	0,0	0,3	0,1	311*
3	4,9	3,2	0,4	0,7	5,9	3,9	0,8	3,2	3,2	11,3	0,9	131	1,9	8,7	8,9	2,2	10,1	0,5	0,4	8,8	2,6	477*	5,9	174
4	1,0	1,1	0,2	0,3	0,6	0,4	0,7	37,1	0,6	0,3	2,2	0,9	0,7	1,5	2,5	1,3	1,9	0,6	0,2	18,9	1,0	4,0	0,6	970*
5	1,3	1,2	0,5	0,3	0,8	0,7	0,5	0,1	0,4	0,5	0,8	0,6	0,7	0,5	0,5	1,3	0,9	0,2	0,3	0,0	1,3	0,1	0,0	0,3
6	3,6	1,6	1,4	0,5	0,7	2,3	1,2	177	1,3	3,0	2,9	88,0	0,8	1,0	2,2	1,5	7,9	6,8	3,1	8,1	0,1	17,4	4,5	110
7	1,4	2,3	0,9	1,0	0,9	2,4	1,0	0,0	1,3	7,4	1,9	1,1	0,7	1,1	1,3	1,2	2,5	1,2	3,8	13,8	0,7	2,8	8,4	0,8
8	2,8	2,1	0,0	0,6	1,6	2,8	3,1	6,6	1,9	8,5	1,8	12,9	39,0	2,2	0,2	3,0	2,8	0,4	0,2	129*	0,7	0,2	8,4	0,0
9	0,5	1,3	1,7	0,4	5,9	1,0	2,2	0,2	0,7	0,4	0,6	0,2	0,4	0,8	0,6	0,5	0,3	0,3	0,3	1,4	0,6	0,0	0,1	0,0
10	0,6	0,3	0,3	0,2	0,8	1,4	0,6	169	0,5	0,4	1,3	0,0	1,1	3,0	0,8	0,6	1,9	0,6	0,5	0,4	64,7	1,8	0,4	71,9
11	1,5	1,3	0,6	1,0	1,0	0,9	1,1	0,3	1,0	1,2	3,4	24,0	1,2	1,7	2,2	1,5	2,6	0,9	0,4	38,9	2,0	6,7	18,0	5,7
12	4,3	1,7	0,8	0,4	0,6	0,6	0,4	0,0	1,5	1,9	3,6	1,3	4,0	1,1	5,2	1,9	3,3	1,7	0,8	29,0	0,9	31,2	16,8	19.1
13	4,1	1,0	2,0	1,7	0,5	1,3	1,5	1,5	0,9	0,5	2,8	3,9	5,7	0,6	1,8	2,1	3,4	11,2	25,3	25,1	10,1	106*	628*	72,3
14	1,1	0,3	0,1	0,3	0,4	0,0	0,1	0,0	0,2	0,2	0,4	2,6	1,9	0,1	0,3	0,6	0,3	0,0	0,0	0,7	0,0	0,1	12,2	0,5
15	3,6	2,2	0,3	0,4	1,6	1,4	1,3	6,2	1,3	1,8	4,0	7,9	2,6	2,0	2,0	2,7	2,3	0,7	2,5	16,0	0,8	0,2	2,4	27,1
16	0,8	0,7	0,3	0,4	0,4	0,6	0,6	0,6	1,8	0,8	0,9	0,8	0,8	1,2	2,9	1,1	0,8	0,2	0,5	15,5	0,0	0,6	0,4	0,6
17	2,3	1,2	0,2	0,5	1,3	2,0	0,9	0,1	0,8	0,9	3,3	1,9	0,8	3,8	2,7	1,6	2,0	0,3	0,4	47,4	0,0	0,8	0,2	25,0
18	1,9	1,8	0,2	0,2	0,2	1,5	0,2	1,2	1,1	1,0	3,0	84,0	0,7	3,4	1,8	1,5	3,9	0,8	0,4	5,3	1,3	40*	0,1	4535*
19	0,8	0,4	0,3	0,3	0,3	0,7	0,6	0,6	0,7	0,5	1,2	10,0	1,1	4,2	3,2	0,7	1,2	0,9	2,8	1598*	0,4	7,9	1172*	633*
20	2,0	2,8	0,6	0,3	0,9	0,6	0,8	0,0	1,0	1,0	3,4	1,9	1,0	0,6	1,3	2,6	1,6	0,5	0,4	3,4	0,5	0,1	0,0	3,1
21	0,5	1,2	0,7	0,5	0,7	1,5	1,0	0,4	0,8	0,7	1,9	1,3	0,9	2,1	1,9	1,6	1,5	0,5	0,8	17,9	0,1	0,1	21,1	8,0
22	0,8	0,8	0,7	0,3	0,1	1,0	0,6	0,0	1,2	2,4	4,3	4,2	1,9	2,8	2,2	1,1	2,3	1,2	2,1	7,8	0,7	0,3	0,1	225
23	0,5	1,3	1,7	0,4	5,9	1,0	2,2	0,2	0,7	0,4	0,6	0,2	0,4	0,6	0,6	0,5	0,3	0,3	0,3	1,4	0,6	0,0	0,1	0,0
24	1,2	1,1	0,4	0,7	0,3	0,3	0,3	1,1	1,1	0,8	1,4	0,3	0,9	1,7	1,2	1,5	1,1	0,4	0,6	0,0	23,3	12,1	29,8	9,2
25	2,6	1,1	0,5	0,6	1,3	0,5	0,5	21,9	1,2	1,7	1,6	1,7	1,1	0,8	1,7	1,1	1,4	0,9	0,7	1,9	1,0	0,0	1,4	1,4
26	1,0	0,7	0,5	1,5	0,2	0,7	0,4	0,0	1,7	1,0	1,3	1,2	4,7	0,4	0,7	0,9	0,8	1,4	2,3	51,5	0,8	34,4	11,0	0,6
27	1,0	0,7	0,1	1,0	0,0	0,4	0,2	27,9	2,5	2,3	3,8	0,7	7,6	1,4	3,4	1,5	2,3	1,5	11,6	730*	0,5	135*	212*	65,1
B. Average T/B fold change in mRNA gene expression of genes upregulated and downregulated in 27 cystectomy patients																								
Number of patients	17	17	4	3	7	11	8	13	13	11	20	19	14	16	19	19	20	8	8	22	8	13	15	19
Average up-regulation	2,47	1,67	1,70	1,50	3,34	1,99	1,72	37,63	1,57	4,08	2,56	20,69	5,44	2,60	2,65	1,69	2,91	3,32	6,69	129,69	13,30	67,51	143,45	382,79
st.dev	1,2	0,6	0,2	0,2	2,4	0,8	0,7	61,5	0,6	3,4	1,0	37,2	9,9	1,9	1,8	0,6	2,2	3,7	8,2	361,9	22,1	129,9	328,1	1036,3
Number of patients	7	8	23	21	19	13	17	14	12	13	7	8	12	10	8	7	7	19	19	5	17	14	12	8
Average up-regulation	0,64	0,60	0,41	0,39	0,50	0,53	0,49	0,13	0,65	0,54	0,69	0,48	0,69	0,60	0,58	0,64	0,63	0,48	0,42	0,33	0,44	0,21	0,19	0,33
st.dev	0,2	0,2	0,3	0,2	0,3	0,2	0,2	0,2	0,2	0,2	0,2	0,3	0,2	0,2	0,2	0,1	0,3	0,3	0,2	0,3	0,3	0,2	0,2	0,3
B2. Average T/B fold change in mRNA gene expression of genes upregulated and downregulated in 27 cystectomy patients. Patient samples identified as outliers by SPSS for respective gene assys have been excluded from the analysis (*)																								
Number of patients	17	17	4	3	7	11	8	13	13	11	20	19	14	16	19	19	20	8	8	22	8	13	15	19
Average up-regulation	2,47	1,67	1,70	1,50	3,34	1,99	1,72	37,63	1,57	4,08	2,56	20,69	5,44	2,60	2,65	1,69	2,91	3,32	6,69	129,69	13,30	67,51	143,45	382,79
st.dev	1,2	0,6	0,2	0,2	2,4	0,8	0,7	61,5	0,6	3,4	1,0	37,2	9,9	1,9	1,8	0,6	2,2	3,7	8,2	361,9	22,1	129,9	328,1	1036,3
Number of patients	7	8	23	21	19	13	17	14	12	13	7	8	12	10	8	7	7	19	19	5	17	14	12	8
Average up-regulation	0,64	0,60	0,41	0,39	0,50	0,53	0,49	0,13	0,65	0,54	0,69	0,48	0,69	0,60	0,58	0,64	0,63	0,48	0,42	0,33	0,44	0,21	0,19	0,33
st.dev	0,2	0,2	0,3	0,2	0,3	0,2	0,2	0,2	0,2	0,2	0,2	0,3	0,2	0,2	0,2	0,1	0,3	0,3	0,2	0,3	0,3	0,2	0,2	0,3
C. Average T/B fold change in mRNA gene expression in aneuploid and diploid patient tumour samples																								
Aneuploid (19 patients)																								
Average	1,9	1,3	0,6	0,6	1,2	1,2	0,8	12,3	1,2	2,1	2,2	13,7	2,0	2,1	2,4	1,4	2,3	1,3	2,9	137	5,7	43	111	325
st.dev	1,4	0,8	0,5	0,4	1,7	0,9	0,5	38,6	0,7	2,8	1,3	34,1	1,9	2,0	1,9	0,6	2,1	2,5	6,0	390	15,3	112	126	1032
Diploid (8 patients)																								
Average	1,6	1,3	0,6	0,6	1,4	1,2	1,2	32,1	1,0	1,9	1,7	17,0	6,0	1,1	1,2	1,3	2,3	1,3	0,9	31,1	0,9	7,9	5,4	137
st.dev	1,0	0,4	0,6	0,5	1,9	0,9	1,0	60,8	0,5	2,8	1,0	29,9	13,4	0,6	0,9	0,8	2,4	2,2	1,2	44,0	0,6	12,3	5,6	339

^*^Gene expression levels identified as outliers by SPSS statistical analysis

#### Gene expression correlation plots

The strength of the correlations of relative mRNA-levels in the different patient samples is visualized in the gene expression signal correlation plots (Fig. 1). The plots display the strength of the correlations between normalised gene expression levels in 15 biopsies of normal bladder mucosa (Fig. 1a), and 27 matched benign/tumour biopsies taken from patients who underwent cystectomy (Fig. 1b and c, respectively). An increasing dissimilarity in gene expression levels and poorer correlations among patients were seen when moving from normal bladder mucosa to neighbouring and tumour tissue.

#### mRNA gene expressions in tumour/neighbouring mucosa from cystectomies compared with normal bladder mucosa

Gene expression patterns (Ct-values) of the normal unrelated mucosa (15 samples) were consistent regarding the two housekeeping genes included in the study. The gene expressions in the tumour and neighbouring tissue collected from the cystectomies were, for all genes included, compared relatively to the gene expression pattern of these 15 samples.

*BMAL1* was down-regulated in both neighbouring and tumour tissue compared to normal mucosa, while *CLOCK* was slightly up-regulated in neighbouring tissue and slightly down-regulated in tumour (Fig. 2).

*PER1* and *CRY1* were both up-regulated in neighbouring and tumour tissue compared to normal mucosa, while *PER2* and *PER3* were up-regulated in neighbouring mucosa and down-regulated in the tumour tissue. *CRY2* was down-regulated in both tissue types compared with normal mucosa. This corresponds well with the immunostaining results (Table 3).

The casein kinases *CSNK1A1L* and *CSNK1E* were down-regulated in neighbouring mucosa and up-regulated in tumour tissue, while *CSNK1A1* was down-regulated in both tissue types (Fig. 2a).

*HRAS* was down-regulated in neighbouring and tumour tissue compared to the normal mucosa, while *NRAS* seemed to be equally down-regulated in neighbouring and up-regulated in tumour tissue. *KRAS*, *EGFR* and *p16* were all up-regulated in both tissue types compared to normal mucosa (Fig. 2b). The tumour suppressors *TP53* and *PTEN* were moderately down-regulated in both tissue types and *uPAR* and *PAI-1* displayed similar patterns. Cytokeratin 1 (*KRT1*) was down-regulated in neighbouring and up-regulated in tumour tissue, while the other cytokeratins (*KRT5-7-10-14*) were all up-regulated in both tissue types compared with normal mucosa. Only *KRT7, PER1, PER2, uPAR, PTEN* and *PAI-1* had p-values below 0.05. This might be explained by the heterogeneity of the patient samples individual gene expression levels, but the tendency described between the biological groups seemed clear.

#### Differences in mRNA gene expression levels in tumour versus benign neighbouring mucosa from cystectomies

Average down- or up-regulation with standard deviation (SD) of each gene expression studied is given in Table 5B. The clock and clock related genes (*BMAL*, *CLOCK*, *PER1*, *PER2*, *PER3*, *CRY1*, *CRY2*, *CSNK1A1*, *CSNK1E*) tended to be either up-regulated or down-regulated from 2-fold to 5-fold in tumour samples compared with matched benign samples. *CSNK1A1L*, which is a homolog to *CSNK1A1*, showed a much higher fold change, from approximately thirty-fold to more than one hundred fold up-regulation in 6 out of 27 patient samples, as well as being not detected and highly down-regulated in a subset of patients. In the majority of the samples, the expression of *BMAL* and *CLOCK* was down-regulated in the tumour tissue compared to matched benign mucosa. *PER1, PER2* and *PER3* were lower in the tumour when compared to neighbouring benign mucosa. For *CRY1,* the gene seemed to be equally up- or down-regulated in the samples, and for *CRY2*, the majority of the samples showed a down-regulation in the tumour tissue. For the three clock related casein kinases, the samples were almost equally distributed between up- and down-regulated gene expression in the tumour tissue, with a wide variation in gene expression levels and hence T/B-ratios.

The mRNA levels for the common tumour markers showed that *p16* was moderately to highly up-regulated in 19 of the 27 samples, while *PTEN* was mainly moderately up-regulated or down-regulated in half the samples each. *TP53*, *EGFR*, *NRAS*, *HRAS*, *KRAS*, *UPAR* and *PAI-1* was generally approximately 2-fold down-regulated or between 2- and 6-fold up-regulated, with some extreme exceptions. The cytokeratins were different from the other genes studied, displaying extremely high T/B-fold changes (100- to 1000-fold up-regulated or highly down-regulated) in subsets of tumours. *KRT1* was mainly down-regulated (17/27 of the samples), while *KRT7* and *KRT14* were mainly up-regulated. *KRT5* and *KRT10* were up- and down-regulated in approximately half of the samples, respectively.

Among the clock genes, the expression of *PER1*, *PER2*, *PER3* and *CRY2* were significantly elevated in the benign tissue compared to the tumour tissue (*p* = 0.001, 0.002, 0.037 and 0.001 respectively) (Fig. 3). The relative quantity of mRNA was significantly elevated in the tumour tissue compared to the benign tissue for *KRT7*, *KRT14*, *NRAS* and *TP53* (*p* = 0.004, 0.010, 0.008 and 0.004, respectively). This also corresponds with Fig. 2b which reveals the same pattern. The expression of *TP53* is lower in the neighbouring mucosa compared to tumour tissue and even more down-regulated in the normal unrelated mucosa. For *uPAR*, the level of mRNA was statistically elevated in the benign tissue compared to the tumour (*p* = 0.019) (Fig. 4), this is also in accordance with the expressions pattern displayed in Fig. 2b.

#### Statistical correlations

Spearman’s rank correlation revealed correlations of the estimated T/B ratios between the various clock-genes. The ones found statistically significant, are listed in Table 6. Statistical significance between the tumour associated genes is listed in Table 7, and correlations between the clock genes compared to other cancer-associated genes are listed in Table 8. The relative quantity of mRNA in tumour compared to neighbouring mucosa was found statistically significant for the genes displayed in Figs. 3 and 4.

**Table 6:** Correlations between the different clock genes

Genes encoding		*p*-value	Correlation coefficient, C
Stimulatory			
*BMAL1*	*- CLOCK*	0.004	0.539
	*- CSNK1A1*	0.003	0.544
	*- CSNK1E*	0.002	0.566
*CLOCK*	*- PER3*	0.001	0.593
	*- CRY1*	0.005	0.522
	*- CRY2*	0.014	0.467
	*- CSNK1A1*	0.029	0.421
	*- CSNK1E*	0.013	0.471
Inhibitory			
*PER1*	*- CRY2*	0.007	0.509
	*- CSNK1A1L*	0.049	−0.382
*PER2*	*- CSNK1A1*	0.001	0.620
	*- CSNK1E*	0.009	0.495
*PER3*	*- CRY1*	0.012	0.475
	*- CRY2*	0.000	0.687
*CRY1*	*- CRY2*	0.000	0.643
	*- CSNK1E*	0.014	0.469
Casein kinases			
*CSNK1A1*	*- CSNK1E*	0.000	0.900

The correlation coefficients: C < 0.3: poor correlation, 0.3 < C < 0.5: fair correlation, 0.6 < C < 0.8: moderately strong correlation and 0.8 < C: Very strong correlation

The genes listed in the right column of the table are found to correlate to the underlined genes in the corresponding left column

**Table 7:** Correlations between the selected tumour markers

Genes encoding		*p*-value	Correlation coeff, C	Genes encoding		*p*-value	Correlation coeff, C
*TP53*	*- PTEN*	0.042	0.395	*HRAS*	*- NRAS*	0.011	0.479
	*- HRAS*	0.005	0.522		*- UPAR*	0.026	0.428
	*- KRAS*	0.000	0.654		*- PAI-1*	0.017	0.455
	*- NRAS*	0.000	0.660		*- KRT7*	0.004	0.536
	*- UPAR*	0.000	0.627		*- KRT5*	0.002	0.569
	*- PAI-1*	0.011	0.482		*- KRT14*	0.000	0.637
	*- KRT7*	0.017	0.455	*KRAS*	*- NRAS*	0.000	0.701
	*- KRT14*	0.011	0.485	*NRAS*	*- UPAR*	0.000	0.627
					*- KRT7*	0.041	0.396
					*- KRT5*	0.001	0.605
					*- KRT14*	0.002	0.561
*P16*	*- KRAS*	0.024	0.432	*PAI-1*	*- KRT7*	0.035	0.407
	*- NRAS*	0.001	0.603		*- KRT5*	0.006	0.518
	*- KRT14*	0.028	0.424		*- KRT14*	0.011	0.479
*PTEN*	*- KRAS*	0.022	0.438	*UPAR*	*- PAI-1*	0.000	0.781
	*- NRAS*	0.047	0.386		*- KRT7*	0.005	0.525
	*- UPAR*	0.008	0.503		*- KRT5*	0.001	0.599
	*- PAI-1*	0.027	0.425		*- KRT14*	0.006	0.519
	*- KRT7*	0.002	0.570				
	*- KRT5*	0.025	0.430				
	*- KRT10*	0.006	0.511				
*EGFR*	*- HRAS*	0.005	0.526	*KRT7*	*- KRT5*	0.017	0.456
	*- NRAS*	0.013	0.471		*- KRT10*	0.017	0.457
	*- KRT14*	0.006	0.511	*KRT5*	*- KRT10*	0.002	0.562
					*- KRT14*	0.002	0.576

The correlation coefficients: C < 0.3: poor correlation, 0.3 < C < 0.5: fair correlation, 0.6 < C < 0.8: moderately strong correlation and 0.8 < C: Very strong correlation

**Table 8:** Correlations between the clock genes and common tumour markers

Genes encoding		*p*-value	Correlation coeff, C	Genes encoding		*p*-value	Correlation coeff, C
*BMAL*	*- TP53*	0.031	0.415	*CSNK1A1*	*-TP53*	0.005	0.527
	*- P16*	0.001	0.601		*- PTEN*	0.001	0.582
	*- PTEN*	0.029	0.419		*- KRAS*	0.004	0.536
	*- KRAS*	0.000	0.670		*- NRAS*	0.000	0.635
	*- NRAS*	0.000	0.694		*-UPAR*	0.001	0.611
					*- PAI-1*	0.003	0.546
*CLOCK*	*- KRAS*	0.000	0.634		*- KRT7*	0.007	0.504
	*- NRAS*	0.005	0.525		*- KRT5*	0.005	0.521
*PER1*	*- UPAR*	0.031	0.416	*CRY-1*	*- P16*	0.040	0.397
	*- PAI-1*	0.047	0.386		*- EGFR*	0.035	0.407
					*- NRAS*	0.001	0.584
*PER2*	*- PTEN*	0.017	0.455	*CSNK1E*	*- TP53*	0.008	0.499
	*- PAI-1*	0.011	0.480		*- P16*	0.013	0.471
	*- KRT7*	0.011	0.480		*- PTEN*	0.021	0.442
	*- KRT5*	0.044	0.390		*- KRAS*	0.008	0.497
	*- KRT10*	0.034	0.409		*- NRAS*	0.000	0.659
					*-UPAR*	0.010	0.487
					*- PAI-1*	0.033	0.412
					*- KRT7*	0.033	0.412
*PER3*	*- KRT5*	0.019	0.448				
	*- KRT14*	0.045	0.389				

The correlation coefficients: C < 0.3: poor correlation, 0.3 < C < 0.5: fair correlation, 0.6 < C < 0.8: moderately strong correlation and 0.8 < C: Very strong correlation

The genes listed in the right column of the table are found to correlate to the underlined genes in the corresponding left column

#### Hierarchical cluster analysis

An unsupervised hierarchical cluster analysis of the relative mRNA-levels was performed and visualized in a heat map (Fig. 5). There were substantial variations between normal mucosal and tumour expression patterns. The neighbouring mucosa exhibited a series of aberrations similar to the tumour and appeared considerably different from the unrelated donor mucosa.

The genes *uPAR* and *PAI-1* clustered and connected to a cluster of *p16* and *KRT7*. Five of the clock genes were also clustered (*PER1*, *PER2*, *PER 3*, *CRY1* and *CRY2*). *CLOCK* clustered with *H-K-N-RAS*, *EGFR* and *TP53*. They clustered with the two cytokeratins (*KRT5* and *KRT10*), which in turn were connected to *KRT14*. The casein kinases *CSNK1A1* and *CSNK1E* clustered and connected to the cluster of *BMAL1* and *PTEN*, whereupon these clusters were connected to the cluster of *CSNK1A1L* and *KRT1*.

Sorted by tissue type, all the normal bladder samples, except for one (11N blue), clustered together. This outlier was placed among the neighbouring samples. There was a similar expression pattern between 6N blue, the outlier, and its adjacent tumour sample (23T red). They seemed to have a lower level of mRNA expression for all genes selected, and all samples in this cluster revealed a low expression of *uPAR* and *PAI-1* (which were strongly correlated; *p* = 0.00, c = 0.781).

The neighbouring samples from the cystectomies were mainly divided into two clusters. In the first, 12 of the neighbouring samples clustered with four tumour samples (5, 7, 9 and 13T). This cluster revealed a lower expression or minor changes in the expression of *BMAL1*, *CLOCK*, tumour marker genes, cytokeratins and casein kinases. The majority of these samples had a higher expression of *PER1*, *PER2*, *PER3*, *CRY1* and *CRY2*. In the second cluster (8 neighbouring samples, 23T and 11N blue), *CLOCK*, *HRAS*, *KRAS*, *NRAS*, *TP53*, *EGFR* and cytokeratin 14, revealed a lower level of expression/minor changes in gene expression. Except for 23T and 11N blue, the neighbouring samples in this cluster also revealed a higher expression of *uPAR*, *PAI-1*, *p16*, *KRT7*, *PER1*, *PER2*, *PER3*, *CRY1* and *CRY2*. Most of the tumour samples accumulated into one cluster (17 samples). One neighbouring sample (14N green) was included in this sub-group. Lower expression of *CLOCK*, the stimulatory clock genes and *PTEN*, together with increased expression of *KRT7* and *KRT14*, characterized this cluster*.* Some aneuploid tumours (15, 17, 19, 21, 22, and 27T) grouped together in a sub-cluster, with increased expression of *HRAS*, *KRAS*, *NRAS*, *TP53* and *EGFR*. A mixed cluster of tumour and neighbouring mucosal samples (normal green: 9, 10, 23, 24; tumour red: 3, 10, 12, 24) revealed higher expression of tumour markers, cytokeratins and casein kinases.

#### Correlations between gene expressions and DNA ploidy

Histological stage and vascular invasion are listed in Table 1. Diploid/aneuploid DNA stemline values are shown in Tables 5C and 9. According to the ploidy of the cancer cells, the average tumour/benign fold change in mRNA levels were similarly expressed for the clock genes except for *BMAL1*, *CRY2* and *CSNK1A1L*. The average expression of *BMAL1* was slightly up-regulated in the aneuploid cells while *CRY2* was slightly down-regulated for the aneuploid cells and up-regulated in the diploid cells. The average for *CSNK1A1L* was up-regulated for both categories, but more than the double for the diploid cancer cells (Table 5C).

**Table 9:** Survey of flow cytometric DNA ploidy in the tumours

WHO grade	Diploid	Aneuploid	Dipl S-phase, mean	Aneup S-phase, mean	Survival
Low	7	3	10.7	16.0	8
High	1	16	25.93	19.55	8

For the other cancer related genes, the total T/B averages for *p16* and *PTEN* were found divergent in the two categories; with four fold higher expression in the diploid compared to the aneuploid stem line. The opposite pattern was seen for *EGFR* and *HRAS*, with an average of two fold higher expression in the aneuploid compared to the diploid cells. The average of the *PAI-1* was slightly down-regulated in the diploid category and almost tree fold up-regulated in the aneuploid cells. Due to individual samples with very high T/B ratios, it was difficult to estimate the cytokeratins’ average in tumour/benign tissue. However, the trend among the five cytokeratins revealed an increased (several T/B-fold) level of gene expression in the aneuploid as compared to the diploid cancer cells.

## Discussion

In the present tumour analyses there were fundamental changes in the cellular clockwork, both as estimated by their gene expression patterns and by immunohistochemistry. The latter parameter not only showed quantitative changes in the tumour cells, but also alterations in the distribution between the nuclei and cytoplasm (Table 3). Several clock genes showed a down-regulation when compared to their own neighbouring mucosa, i.e. *PER1*, *PER2* and *PER3,* while *CRY2* was down-regulated in both tumour and neighbouring tissue when compared to normal mucosa from unrelated donors (Fig. 2a). In contrast, *PER1* and *CRY1* were up-regulated in tumour and neighbouring mucosa compared to the normal donor tissue. These findings were consistent with the IHC data (Table 3). One of the casein kinases (*CSNK1A1*), which is known to have a critical regulatory role in transmitting signals from the clock genes, was reduced [27].

We also found a moderately strong correlation between the T/B ratios of *PER2*, *CSNK1E* and *CSNK1A,* respectively (Table 6). When using the neighbouring mucosa as reference to tumour, the picture became complex, since the mucosa may already have acquired preneoplastic properties or different influences from malignant tissue. It was striking that clock gene aberrations were found mainly in aneuploid tumours of high grade. The same applied to increasing heterogeneity in tumour as well as neighbouring mucosa.

When compared to normal unrelated mucosa, all the cancer related oncogenes except *HRAS*, were strongly up-regulated, while the two suppressor genes *TP53* and *PTEN* were down-regulated (Fig. 2b). In line with other studies [28], we have earlier reported that there is an accumulation of the p53 protein in these tumour cells, possibly a non-functional suppressor protein, while PTEN seems to be largely absent [25, 29]. The strong up-regulation of high molecular weight cytokeratins found in the gene-expression analysis is also consistent with our earlier findings [25]. The accumulation of these proteins has been related to a worse prognosis. The same relates to an up-regulation of the plasminogen activator (uPAR) and the inhibitor, PAI-1, which at high levels paradoxically stimulates invasive growth.

PAI-1 expression in different tissues is closely controlled by clock genes *in vivo*. Loss of clock genes may result in an increased PAI-1 expression and constitutes a contributing risk factor for cardiovascular disease. There is also a possibility that CRY suppresses *PAI-1* expression independent of its clock function. It has been suggested that clock genes and RAS may differentially affect the circadian expression of PAI-1 in various tissues. Other studies reveal that the basic helix-loop-helix (bHLH)/PAS domain transcription factor plays a crucial role in controlling the biological clock that control the circadian rhythms. In line with this, a novel bHLH/PAS protein cycle-like factor (CLIF) regulates the circadian regulation of PAI-1 gene expression in endothelial cells [30, 31].

A surprising finding was that oncogene overexpression was both correlated to the levels of stimulatory and inhibitory clock genes (Table 8). We have earlier reported a strong up-regulation of EGFR and p16, including H-, K- and N-RAS [25, 29], and in the present mRNA analysis all of these tumour genes were strongly correlated (Table 7 and 8). This extends earlier findings that malignant behaviour in urothelial cells may at least in part be due to a combined action of oncogenes, altered suppressor genes and aberrant clock gene expression [32–34]. However, the present data do not give any information with regards to which of these three gene classes is the primary cause of this deviation. Alternatively, mutations and/or deletions in either of them, leading to non-functional proteins could be critical steps in development of biological malignancy.

The finding that such combined aberrations are almost exclusively in high grade, aneuploid tumours, points in the same direction. Thus, it has been known for several decades that aneuploid urothelial cancers have a higher malignant potential, accompanied by a higher frequency of aneuploidy in the neighbouring normal appearing mucosa [26]. The expressions of uPAR and PAI-1, which mediate a cascade of other cellular functions related to invasiveness and proteolysis, were also correlated to alterations of the clock gene expression points in the same direction (see Table 8).

In rodents, it has been reported that mutation of the *CSNK1* priming site in *PER2* (Ser662), leads to decreased phosphorylation of stabilizing sites in *PER2* and accelerated circadian rhythms. PER1 and 2 have the highest amplitude oscillations of all the known core clock proteins, with almost complete degradation near the end of the subjective night in the SCN. PER2 also undergoes temporal changes in phosphorylation that reaches a zenith just prior to its destruction. Both kinase/phosphatase activities are thought to regulate PER2 net phosphorylation and stability [35–37]. PER2 has also been found to function as a tumour suppressor, with the absence of both its copies causing an increased rate of radiation-induced cancers. It now seems evident that its anti-cancer action arises from the ability to turn off Myc. In the absence of PER2, Myc levels greatly rise, thereby explaining why many types of tumours display higher levels of CSNK1E than their normal cell equivalents [38].

The majority of all advanced human tumours have mutations in the *TP53* gene, and in rodents *PER2* expression is also found directly regulated by p53 binding to a response element in the *PER2* promoter. This p53 response element is evolutionarily conserved and overlaps with the E-Box element critical for BMAL1/CLOCK binding and its transcriptional activation of *PER 2* expression. In consequence, p53 may block BMAL1/CLOCK binding to the *PER2* promoter, where the cellular level of PER2 is inversely correlated with that of p53. Studies also suggest that functional PER2 is important for p53-mediated stress signals to reach the circadian clock network and that p53 acts as a transcription factor that regulates the circadian clock by direct control of *PER2* expression [39]. A common paradox is that that there may be an accumulation of the protein in malignant cells in spite of their unrestricted growth. Our observation of down-regulation of the gene expression combined with accumulation of p53 in urothelial cancer (Fig. 2) is therefore a common finding in these tumours [25, 40]. Surprisingly, the reduced transcription of the tumour suppressor p53 was correlated to the expression of clock genes and related casein kinases (Table 8).

Since we have only investigated cystectomies and unrelated normal mucosa harvested in the first part of the light period, i.e. before noon, high transcriptional levels of the inhibitory genes and low levels of the stimulatory ones would be expected. As shown in our Results, this was not the case, indicating a disturbance of the circadian timing in the malignant urothelial cells. However, two open questions remain: Could the observed clock gene alterations be due to a longstanding phase shift of otherwise normal oscillations and not a disruption of the clock work *per se*? Although our data do not warrant a firm conclusion on these questions, they mainly suggest a severe perturbation of the cellular clocks. One can speculate whether the preparation for surgery and surgery/anesthesia itself might lead to differential disruption of endogenous circadian homeostasis in both normal and tumour tissue. However, all the patients included in our study are diagnosed with bladder cancer and have undergone the same surgical procedure. Corresponding studies of clock genes in human tissues used for comparison are also conducted on tissue harvested from surgical specimens. The close relation of these changes to the up-regulation of other cancer-associated genes also indicates a disruption of the clock. Since sequence sampling of cancerous tissue fragments and cells for investigating the whole circadian cycle is at present not clinically possible, a final answer remains open.

Since biological markers for bladder cancer reported so far have only been of limited clinical value [32], clock gene markers might therefore serve as an adjunct to other diagnostic and prognostic histological/biological markers. The present study has some limitations with respect to the number of cases included, which hence affects the statistical power and makes it difficult to draw a broad conclusion. However, the strong significance between several independent parameters makes it unlikely that this is due to random variations. Future expanded studies are warranted to validate the role of the different markers selected in this study.

## Conclusions

A correlation was found between altered mRNA and protein expression of various clock genes and common tumour markers in urothelial cancer, indicating that disturbed function in the cellular clockwork may be an important additional mechanism contributing to cancer progression and malignant behaviour. These alterations are most pronounced in aneuploid, high grade tumours, and are to some extent also seen in the neighbouring mucosa.

## Abbreviations

A, aneuploid; A-S, Aneuploid S-phase; B, benign tissue; bHLH, basic helix-loop-helix; BPH, benign prostatic hyperplasia; C, correlation coefficient; CLIF, cycle-like factor; D, diploid; D-S, Diploid S-phase; FCM, flow cytometry; G, grade; N, normal tissue; PI, ploidy index; qPCR, quantitative polymerase chain reaction; r, Pearson’s product monument correlation coefficients; RQ, relative quantity; SEM, standard error of mean; Survival A/D, survival after surgery; T, tumour tissue; TLDA, taqman low density arrays; TUR-P, transurethral resection of the prostate; V.I., vascular invasion

## Additional file

Additional file 1: Figure S1. Immunohistochemical staining of various clock proteins in urothelial cancer as compared to normal controls stained in parallel. Counterstained with hematoxylin (blue nuclei). Magnification is given by the tool bar: 10 μm. A: PER1 in tumour showing the same staining density as in controls (B). C: PER3 with positive nuclei in tumour as compared to negative in the controls (D). The cytoplasm is slightly positive in both. E: CRY1 with increased positivity in tumour cell cytoplasm as compared to the control (F). G: CRY2 with negative reaction in tumour cell nuclei and positive in controls (H). I: BMAL-1 with approximately equal staining in tumour cells and control (J), i.e. moderate staining in nuclei and strong in cytoplasm. For semi quantitative estimates and details, see Table 3. (PDF 617 kb)
