# Supplementary material for: Expression of circadian clock genes and proteins in urothelial cancer is related to cancer-associated genes
Source: BMC Cancer. 2016 Jul 27;16:549. doi: 10.1186/s12885-016-2580-y (PMC4964027; doi:10.1186/s12885-016-2580-y)

Additional files

Additional file 1; Legend to illustration:

Figure S1. Immunohistochemical staining of various clock proteins in urothelial cancer as compared to normal controls stained in parallel. Counterstained with hematoxylin (blue nuclei). Magnification is given by the tool bar: 10µm. A: Per1 in tumour showing the same staining density as in controls (B). C: Per3 with positive nuclei in tumour as compared to negative in the controls (D). The cytoplasm is slightly positive in both. E: Cry1 with increased positivity in tumour cell cytoplasm as compared to the control (F). G: Cry2 with negative reaction in tumour cell nuclei and positive in controls (H). I: Bmal-1 with approximately equal staining in tumour cells and control (J), i.e. moderate staining in nuclei and strong in cytoplasm. For semi quantitative estimates and details, see Table 3.

Figure S1

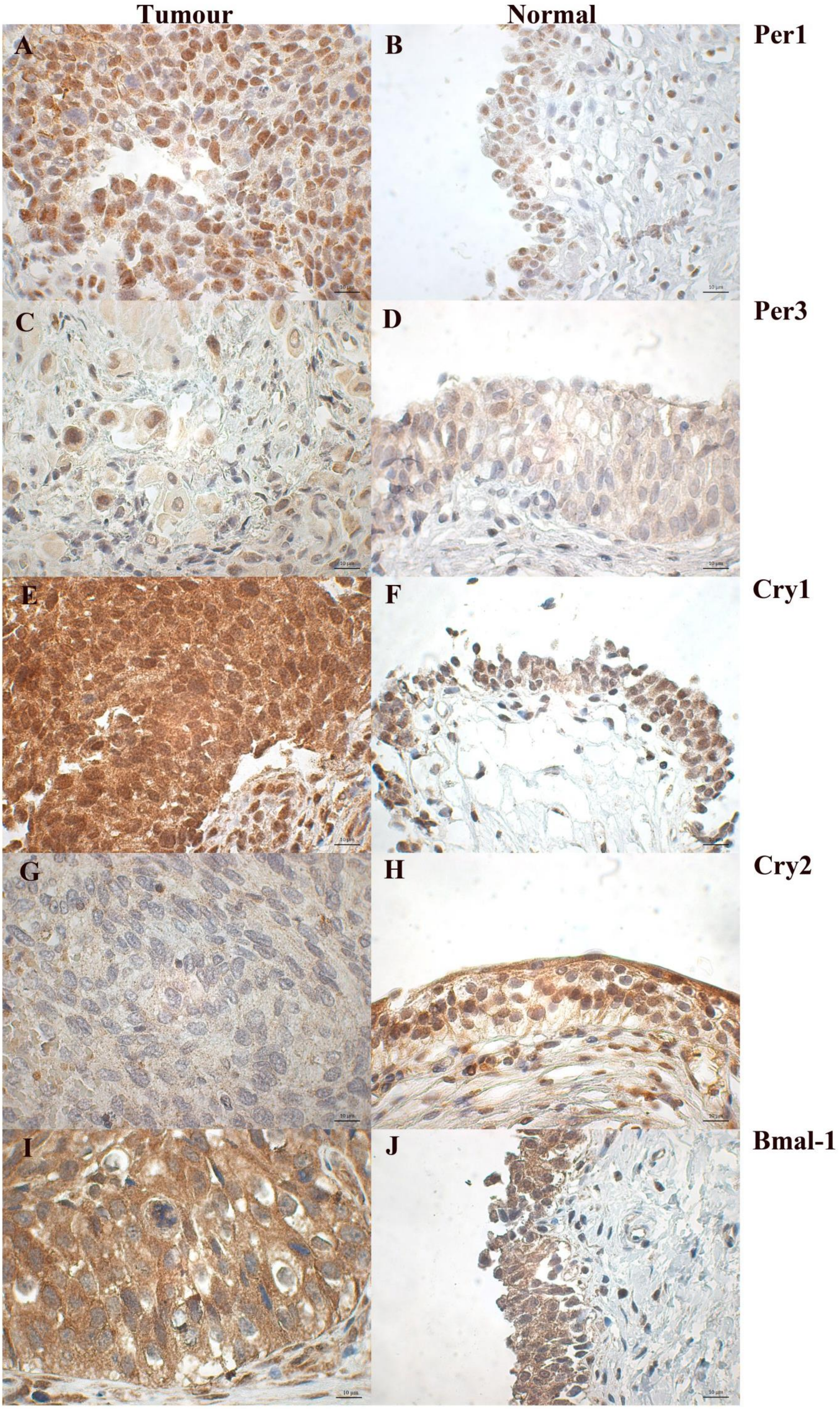

Supplement: Additional file 1: Figure S1. — Immunohistochemical staining of various clock proteins in urothelial cancer as compared to normal controls stained in parallel. Counterstained with hematoxylin (blue nuclei). Magnification is given by the tool bar: 10 μm. A: PER1 in tumour showing the same staining density as in controls (B). C: PER3 with positive nuclei in tumour as compared to negative in the controls (D). The cytoplasm is slightly positive in both. E: CRY1 with increased positivity in tumour cell cytoplasm as compared to the control (F). G: CRY2 with negative reaction in tumour cell nuclei and positive in controls (H). I: BMAL-1 with approximately equal staining in tumour cells and control (J), i.e. moderate staining in nuclei and strong in cytoplasm. For semi quantitative estimates and details, see Table 3. (PDF 617 kb) [file 12885_2016_2580_MOESM1_ESM.pdf]
